# Deciphering obesity: a conceptual overview and synthesis

**DOI:** 10.3389/fendo.2025.1738147

**Published:** 2026-01-07

**Authors:** Rashmi Prakash, Anshu Arora, Arif Khan

**Affiliations:** 1 Northwest Clinical Research Center, Bellevue, WA, United States; 2 Pacific Northwest University of Health Sciences College of Osteopathic Medicine, Yakima, WA, United States

**Keywords:** epigenetics, hunger, incretins, metabolic memory, obesity, satiety

## Abstract

Obesity represents a multifactorial disorder rooted in genetic, developmental, and environmental influences. Its persistence and heterogeneity warrant deeper understanding of pathology beyond genetic determinism. This Perspective synthesizes a conceptual model that draws from fundamental concepts, establishes cellular and organ levels of pathology, and provides a functional hierarchy of molecular signals in energy regulation. At the cellular level, DNA, RNA, and epigenetic regulators act as scripts, playwrights, and directors shaping metabolic behavior, establishing a “metabolic memory” that influences lifelong vulnerability, whereas peptides, proteins and other functional molecules are the actors. At the organ level, a four-organ model of hunger—the stomach (initiator), brain (executor), pancreas, and gut (regulators)—explains how hunger arises as a transient deviation from satiety, the body’s homeostatic baseline. These organs communicate through rapid neural and slower chemical routes, integrating homeostatic and hedonic signals of food intake. At the molecular level, signaling molecules can be organized by size and function: small neurotransmitters that perform rapid communications, medium-sized peptides (ghrelin and incretins) that denote the messages to be conveyed and larger molecules like leptin that establish long-term tone. Recognizing this relationship clarifies why incretin therapies, though transformative, remain symptomatic rather than curative. These insights highlight the need for therapies that reprogram metabolic memory and restore durable equilibrium. Integrating these insights in research and practice would align obesity management with its true biological complexity.

## Introduction

Obesity prevalence has nearly tripled globally in the past century, paralleling advances in food production and availability. Though classified as a disease, debate continues over whether obesity is intrinsically harmful or an expected human variant in food-abundant environments (1). Some with higher body mass remain metabolically healthy, while others develop diabetes or cardiovascular disease at lower weights. The challenge lies less in aesthetics and more in the physiological consequences of changing diets and excess adiposity, namely metabolic strain, reduced quality of life, and increased disease burden (2).

Genetics are often invoked as intractable determinants of body weight. While rare monogenic and syndromic forms like melanocortin or leptin-receptor mutations and Prader–Willi syndrome illustrate clear genetic causation, most of today’s epidemic does not arise from isolated mutations. Family and twin studies demonstrate high weight heritability not sufficiently explained by the numerous genetic variants (e.g. FTO gene) identified in genome-wide studies; protective variants, such as in the GPR75 gene, also exist (3). Importantly, human DNA has not changed substantially in the brief period during which obesity prevalence has surged. Therefore, genes alone cannot explain the epidemic. Describing obesity as a “polygenic” disorder risks a defeatist outlook and neglects the interpretive machinery linking genotype to phenotype.

Categorizing obesity as a chronic disease indicates pathology involving multiple organ systems across the lifespan (4). Other chronic diseases like hypertension have diverse drug classes targeting distinct but complementary mechanisms—e.g., angiotensin receptor and calcium channel blockade. Multiple drugs are often needed to achieve individual therapeutic goals (5). However, currently approved anti-obesity drugs are fewer and narrower in scope (6).

More recently, incretin-based molecules like semaglutide and tirzepatide have markedly advanced obesity management. Peripherally, they delay gastric emptying, prolong fullness, increase fat oxidation and promote energy expenditure, whereas their central mechanisms in obesity, still under study, are on neural satiety pathways (6, 7). However, limitations like high costs, adverse effects, and discontinuation rates constrain real-world effectiveness (7). Responses vary between individuals, effects may plateau, and weight frequently returns after discontinuation; a minority of patients fail to achieve meaningful benefit despite adherence (8). The large effect sizes of these drugs distract from the fact that they are not beyond symptom-reduction. Despite a growing therapeutic pipeline, reliable response predictors and durable metabolic remission remain elusive (7, 8**).**

The above gaps in the understanding and management of obesity indicate that its foundational elements may not yet be fully integrated into current clinical and research approaches, thereby placing undue responsibility on patients without actionable strategies. These questions must frame how we think about current and future therapies.

In this article, we propose a unifying model of obesity that integrates and organizes the underlying mechanisms. Our model integrates fundamental concepts from cellular and developmental biology with organ-level physiology of body weight, detailing how they shape individual vulnerability. The model reframes hunger as a discrete deviation from satiety mediated by a four-organ circuit, and introduces a functional hierarchy of molecules involved in weight regulation, that relates their size and temporal durability. This explains why approved obesity pharmacotherapy does not yield lasting effects. We then consider how it can inform current and future research and clinical care.

By consolidating established concepts into a clear structure, the model is intended not as a comprehensive review but as a tool to prompt re-evaluation of prevailing assumptions and to encourage a broader view of obesity.

## Overview of concepts

The central dogma of genetics traditionally describes a linear path from DNA to RNA to proteins. However, “junk” RNA subtypes like microRNA and long non-coding RNA (lncRNA), previously thought to not yield functional products, are now recognized for their regulatory role (9). Along with DNA-modifying enzymes like methyltransferases and histone modifying proteins, they determine DNA expression without altering its physical structure. This domain, called epigenetics, governs development and cell differentiation, and is dynamic, adapting to internal and external environments (3, 9).

At this juncture, we consider the pre-DNA work of Alan Turing (10), which describes the physical and chemical factors during development resulting in morphogenesis. Chemicals called morphogens develop concentration gradients, which direct cell migration and differentiation, break symmetry and produce variations arising from a homogenous system. This theory, subsequently supported by evidence (11), explains why genetically similar individuals diverge in phenotypes, including traits like weight and food-related behaviors.

Complementing this is the extensive work of Barker and colleagues, who observed that individuals exposed to food scarcity during gestation and childhood had higher risks of developing obesity, diabetes and hypertension in adulthood (12). Thus, the Developmental Origins of Health and Disease (DOHaD) theory took form, which posits that early life environments play a crucial role in how the body interprets varying energy needs and food availability throughout life, a phenomenon termed metabolic programming (13).

This concept extends into adulthood, aligning with what is now termed metabolic memory or “legacy effect”. Diabetes research shows that cumulative effects of chronic hyperglycemia continue to increase risks of diabetes complications despite glycemic normalization; early and intensive glycemic control reduces long-term risks (14). Similar phenomena have been observed in obesity. Epigenetic alterations caused by diet-induced obesity in mice and humans were found to persist after significant weight loss (15). Further, these epigenetic patterns were heritable across generations. This helps explain the tendency to regain weight after weight-loss interventions, weight-cycling and yo-yo dieting, and adds to existing knowledge of weight heritability.

We now view these concepts in an evolutionary context. Human metabolism evolved in intermittent food availability; fasting periods were longer and more frequent than feeding. The energy-replete state or satiety was therefore the body’s equilibrium, while hunger arose as a transient, adaptive perturbation, signaling the need to restore balance. Energy homeostasis seems to have thus evolved to preserve satiety efficiently and not to sustain constant feeding; modern food-abundant environments have overwhelmed this system (16).

We hereby defer to the satiety cascade proposed by Blundell and colleagues (17). They described the key components of appetite: hunger, satiation (meal termination), satiety (fullness or satisfaction), liking (sensory pleasure) and wanting (motivation to seek) of food. This model explains that eating behavior is governed by physiological, neurobiological, and psychological factors. Physiological factors relate to metabolic needs, neurobiological processes involve brain activity underlying hunger and satiety, and psychological factors include cognitive and sensory processes influencing food-related behavior, e.g., hunger perception and cravings.

The recently described “food noise” phenomenon adds to this (18). Individuals with obesity who lose weight on incretin-based therapies often describe mental quietness, noting the lack of preoccupations around food and food choices. This, alongside the demonstrated benefits of these therapies in psychiatric conditions like substance abuse, binge-eating disorder and depression (19), indicates that they influence neuro-behavioral aspects of food intake, which are often unaddressed in clinical practice.

We now describe a theoretical framework for obesity that consolidates these concepts at cellular, molecular and organ-system levels.

## Cellular level: 

**Figure 1:**
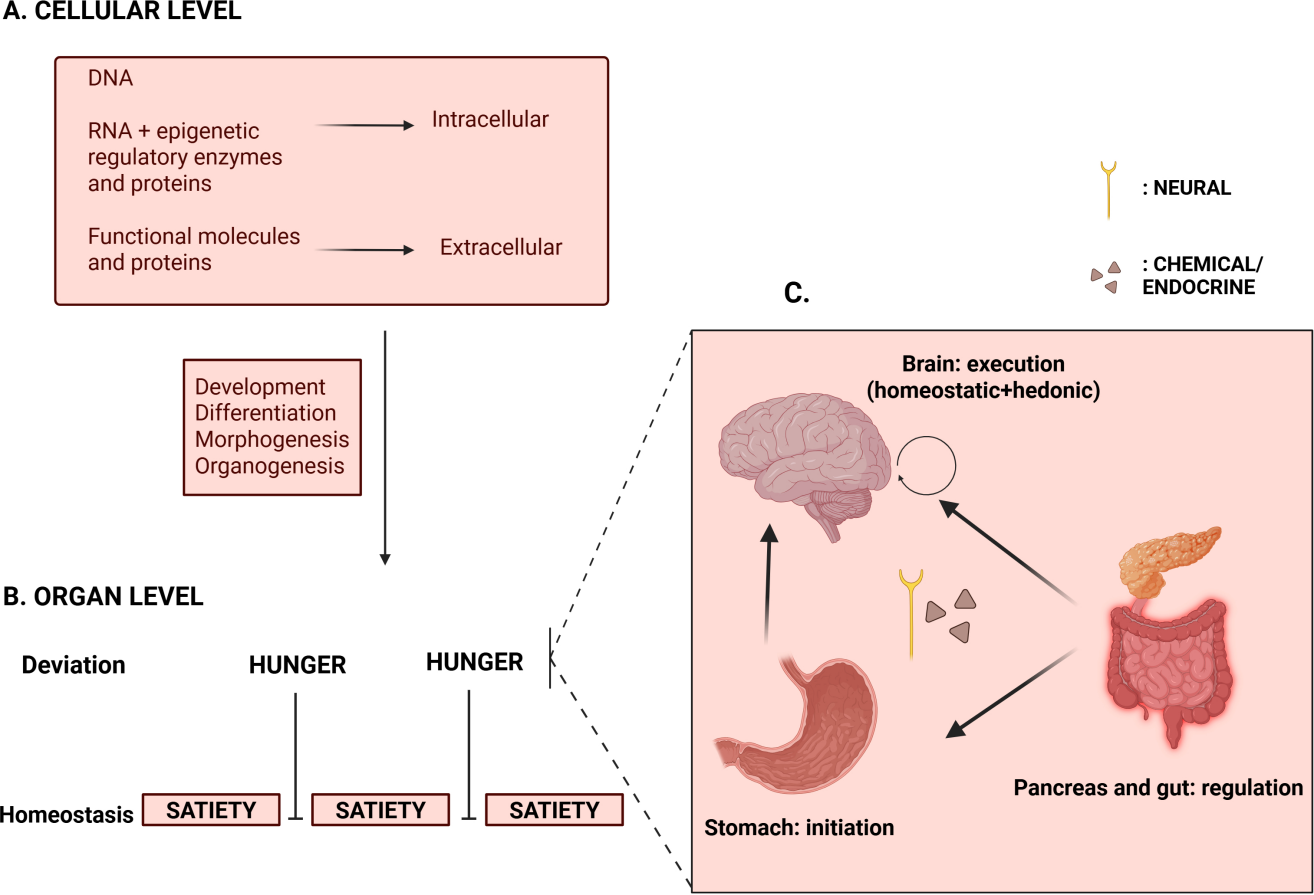
**(A)** Cellular level of regulation: intracellular and extracellular molecules perform specific functions in integrating genotype to phenotype. Various factors act during the development of cell into an organ; these factors determine how genetic scripts are interpreted. **(B)** Organ level: Hunger emerges as a deviation from the homeostatic equilibrium of satiety. **(C)** Four-organ model of hunger: Physiological or homeostatic hunger is initiated by the stomach. This is integrated with hedonic or non-homeostatic influences at the brain, which executes them into food-seeking behavior. Regulation of hunger and return to the baseline of satiety is mediated by the pancreas and gut through their various peptides, proteins and other molecules. Created in BioRender. Prakash, R. (2026) https://BioRender.com/rioa8re.

(**Figure 1A**) We analogize DNA as the script, RNA and epigenetic regulatory enzymes as the playwrights and directors, and functional molecules like proteins as the actors who perform according to internal and external environments. The early zygote depends on pre-packaged maternal RNA, enzymes and proteins until about four to eight-celled stages after fertilization. It then becomes autonomous (i.e., zygotic genome activation) (20). Subsequently, the blastocyst implants into the endometrium; morphogenesis and organogenesis proceed. Several factors affect development throughout. Chemical factors include maternal metabolism, nutrient availability for synthetic and metabolic functions of the developing fetus, blood supply, toxins and infections. Physical factors like morphogen gradients, site of implantation and cellular orientation within the blastocyst influence nutrient availability and utilization. Epigenetic mechanisms of the developing fetus adapt and function accordingly.

As the embryonic cells become more specialized and differentiate into various organs, they begin performing more specific functions relating to energy intake, storage, sensing, and expenditure. Thus, body weight emerges as a function of these processes.

## Organ level

From human evolutionary feeding profiles, we regard “satiety” as the homeostatic baseline. Physiological energy needs cause periodic deviation from the baseline in the form of hunger (**Figure 1B**). To consolidate the origin and termination of this deviation, we frame hunger (21) as a function of four organs (**Figure 1C**): the stomach (initiator), the brain (executor), the pancreas and the gut (regulators). Inter-organ communication is achieved through a rapid neural (vagal) route, and a slower chemical route, mediated by the ‘actors’, namely peptides and proteins produced by the respective organs.

Stomach emptying and shrinkage lead to a loss of stretch-receptor stimulation on vagal afferents innervating it. This sets off the rapid neural signals to the brain. Falling nutrient levels trigger ghrelin (“hunger hormone”) secretion, which is the chemical signal to the brain (21).

These signals are consolidated within brain centers, notably brainstem and hypothalamus. Non-homeostatic, or hedonic factors, i.e., liking and wanting of food, are mediated by limbic and reward circuits, and influence food intake, often independent of physiological signals of hunger, satiation and satiety. These are shaped by social and emotional contexts—e.g., one would delay food intake to an appropriate time and place like lunch break, or could overeat beyond satiation (17, 21). Thus, cognitive and hedonic processes may significantly override homeostatic signals.

Communication between brain regions, e.g., hypothalamus and the cortex, occurs through neuropeptides like neuropeptide Y (NPY), orexin and Agouti-related Protein (AgRP), and smaller neurotransmitters, like Gamma Amino-Butyric Acid (GABA), glutamate and dopamine (17, 21, 22). Brain neurotransmission leads to execution of goal-directed motor activity, i.e., behavior, at the cortex and basal ganglia, leading the individual to seek and eat food (21).

As food becomes available, pancreatic insulin secretion, which starts at the cephalic phase of digestion acts as the first hunger regulator. Stomach filling and nutrient availability activate stretch and inhibit ghrelin, terminating hunger signals. Food then moves through the gut, which acts as the second hunger regulator via gut distension(neural) and gut peptides (chemical, e.g., cholecystokinin—CCK, incretins and Peptide YY), which signal satiation to the brain, leading the individual to stop eating food. Central satiety regulation involves neuropeptides like alpha-Melanocyte Stimulating Hormone (αMSH) and neurotransmitters like serotonin (17, 21, 22).

Beyond hunger and satiation, a larger network of organs governs the subsequent events of satiety, energy storage and expenditure. The adipose tissue provides tonic energy availability signals through adipokines like leptin and adiponectin, sustaining inter-meal satiety (21, 23). The gut microbiome contributes to satiety through its metabolites like bile acids and short chain fatty acids (SCFA) that maintain gut epithelial and immunological integrity (23). Energy may be utilized or stored based on need dictated by requirements of the various components of energy expenditure (i.e., resting metabolism, physical activity and digestion) (24). The liver and skeletal muscle produce proteins like fibroblast growth factor 21 (FGF21) and myokines, which facilitate energy expenditure (23–25). Nutrient sensing through specialized receptors occurs throughout, guiding how signals of energy need and availability are generated and interpreted (26).

These processes shape the physiological landscape within which hunger occurs. However, we retain our focus on hunger as it is the most behaviorally consequential internal cue that translates physiology into action, and that is predominantly targeted by approved obesity interventions (6).

## Molecular hierarchy

In reality, these processes do not occur in clear-cut sequences, but in parallel, influencing one another in multiple directions. However, this model provides a didactic scaffold and uncovers various pathways and molecules that could become pharmacotherapeutic targets. Disruptions in any of these pathways, regardless of source, cause metabolic and immunological strain (27). For example, excess adipose tissue deposition outpaces angiogenesis, leading to adipocyte hypoxia and increased pro-inflammatory macrophage activity (27). Dietary saturated fats produce selection pressure within the gut microbiome, altering bacterial populations. This dysbiosis alters antigenic load and gut epithelial integrity, contributing to inflammation. Dietary saturated fats and inflammatory mediators disrupt second messenger signaling at leptin and insulin receptors both centrally and peripherally (26, 27). These events lead to disrupted signals of energy need and availability, extending into a systemic, self-perpetuating loop of chronic low-grade inflammation and weight gain. Similarly, targeting one molecule would have cascading effects on the rest of the loop.

This leads us to wonder if obesity is conceptually analogous with autoimmune diseases that have diverse manifestations, and require lifelong immunomodulation through different molecular targets. However, before we lose ourselves in this complex web of molecules, we call back to the concept of scripts, playwrights, directors and actors. Would targeting an actor cause any lasting change in the play, or simply disrupt workflows without touching biological roots?

**Figure 2:**
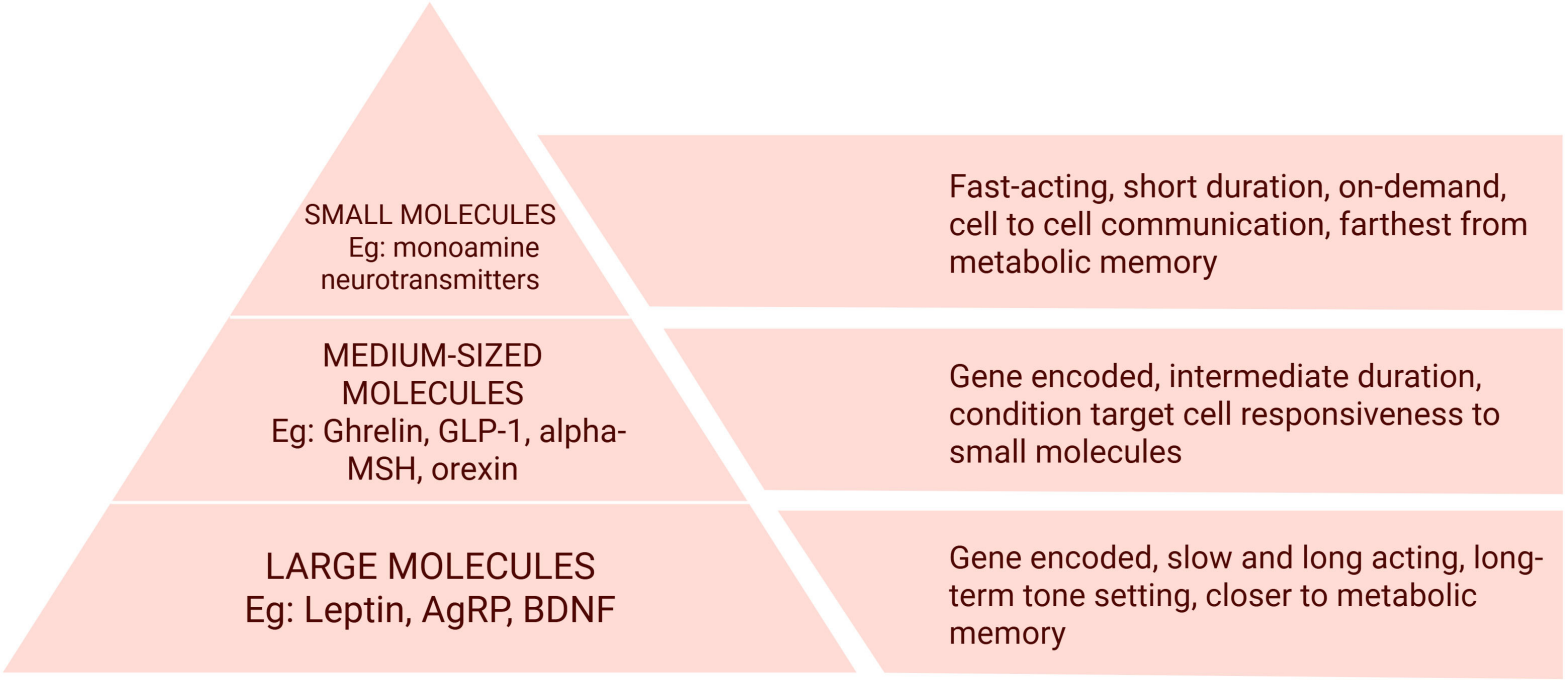
The molecular hierarchy of extracellular actors: smaller molecules like neurotransmitters are short-acting messengers and bring about immediate cell-to-cell communication. Medium-sized molecules condition cells and determine the message that needs to be conveyed based on internal and external environments. Larger molecules, which act for longer set the baseline context in which the other two categories of molecules operate. GLP-1, Glucagon-like Peptide-1; alpha-MSH, alpha-Melanocyte Stimulating Hormone; AgRP, Agouti-Related Protein; BDNF, Brain-Derived Neurotrophic Factor. Created in BioRender. Prakash, R. (2026) https://BioRender.com/rioa8re.

Hence, we suggest that achieving durable remission in obesity warrants efforts to reprogram metabolic memory. To frame this concept, we draw on the observation that larger extracellular molecules act for longer durations and interface more directly with intracellular processes that encode metabolic memory (9, 13–15, 21), than smaller ones. We thus propose a functional size-based hierarchy of molecules that mediate body weight, behavior and metabolism (Khan et al., manuscript under review, 2025), based on the temporal durability of their actions rather than their biochemical structure (**Figure 2**).

Firstly, with the intracellular scripts and actors, we include intracellular proteins involved in the synthesis, metabolism and transport of the extracellular actors. These intracellular molecules mediate the actions and effects of extracellular ones, and chronicle physiological events into heritable memory (9).

Among the extracellular actors, the smallest molecules are neurotransmitters, which act quickly and transiently to effect neuronal communication and behavior. Glycine, choline, histidine, tyrosine, and tryptophan, with their low molecular weight derivatives, namely glutamate, GABA, acetylcholine, histamine, catecholamines and indoleamines, perform cell-to-cell communication within the brain and translate physiological, cognitive and psychological processes into behavior (28).

Next, medium-sized molecules, roughly fifty amino acids in length, like ghrelin, Glucagon-like Peptide-1(GLP-1), αMSH, etc. determine the message that needs to be communicated by neurotransmitters. They are larger and need a longer time for synthesis, storage and release; they have longer durations of action at their receptors. They condition target cells, modulating neurotransmitter release and responsiveness. For example, GLP-1 activates its receptors on vagal afferent terminals in the intestine, triggering action potentials that relay through the nodose ganglion at the skull base, and leads to excitatory neurotransmission in brain satiety pathways (21, 28).

Lastly, we have the largest extracellular actors, like leptin, AgRP and brain-derived neurotrophic factor (BDNF; involved in neuroplasticity, the brain’s ability to adapt structure and function in response to intrinsic and extrinsic stimuli) (29). They sustain the signals conveyed by small and medium molecules. They operate more slowly, influencing cellular metabolism, gene expression, and receptor availability. Therefore, they reinforce long-term metabolic tone and define the enduring “climate” within which signals are interpreted (29).

Thus, it is clear why GLP-1 analogs do not alter the course of obesity beyond the treatment period. Neurotransmitters and medium-sized molecules, though critical for inter-organ communication, cannot fully account for the persistence or depth of metabolic disorders.

## Discussion

### Research implications

This perspective helps clarify why obesity remains pervasive despite large investments and advances. It explains why physiological disruptors do not produce universal effects; for example, monozygotic twins can have different weights and eating behaviors despite similar genes and environments (30). It also explains why incretin-based therapies are not the final word.

Whereas humans could take on an evolutionary course dictated by food overabundance, unlike the apocalyptic Malthusian predictions, it is unhelpful to wait for evolution to catch up (16**).** In a food cue-rich environment, vulnerable individuals need sustainable ways to protect against need-dissonant eating behavior.

The serendipitous success of incretin molecules provides a window into deeper levels of obesity pathology where individual vulnerability is rooted; the larger extracellular proteins and intracellular molecules represent potential disease-modifying targets to reprogram metabolism. Currently, several molecules are being studied (31); RNA-based therapies targeting fat metabolism genes is one example (31). Although in its infancy, intracellular and large molecular targeting hold considerable promise.

Whereas current obesity therapies target brain neurotransmission, (naltrexone-bupropion, phentermine-topiramate), satiety pathways (incretin-based therapies) and intestinal fat absorption (orlistat) (6), we believe in exploring the larger network of organs and processes in obesity, like nutrient sensing, gut microbiome and adipocyte inflammation for potential targets at the small to medium-sized molecular level.

Obviously, a one-size fits all approach would not be practical. Indeed, past attempts at replacing leptin had almost no effect in obesity without genetic leptin deficiency (32). Therefore, we believe that identifying, and not assuming, the origin-point of metabolic disruption within the vicious loop (e.g., targeting leptin resistance instead of replacing leptin in “polygenic” obesity) (32), would help improve therapeutic outcomes. These concepts form the core of phenotypic classifications of obesity and precision medicine (33). Research is ongoing to identify molecular biomarkers of obesity in therapy and prevention, such as interleukin-6 (IL-6) and FGF21 (33, 34).

### Practical implications

The physician’s dilemma remains: those seeking solutions for excess weight are often adults, looking for results within their lifetime. We can neither alter early-life exposures that have already occurred, nor currently modify inherited scripts of obesity.

However, we can focus on early identification of factors that offer patients an immediate entry point for durable change. Identifying risk factors in eating behavior is an example. For instance, food noise has been reported across individuals with varying degrees of adiposity, suggesting that it reflects a neurocognitive phenomenon that may precede weight gain. It may thus serve as a point of potential intervention (18), though empirical evidence on its temporal sequence is needed. Other modifiable risk factors not involving polygenic risks are psycho-social stressors, comorbid medical, psychiatric and neurodevelopmental conditions, and altered circadian rhythms (e.g., shift-work disorder). These factors influence treatment adherence and response and thus, must be evaluated in practice (35).

While research builds on the above model to establish durable interventions, attention must be turned to clinician training. As it stands, current pharmacotherapy is not yet the intuitive first-line in primary care (36), nor has the classification of obesity as a disease fully caught on; clinicians continue to rely on lifestyle education, shift responsibility and blame onto patients, and reserve pharmacotherapy and metabolic-bariatric surgery (MBS) for patients with very high body mass index (BMI), or with comorbidities like diabetes (37). Focused efforts to move providers toward early medical intervention and multi-disciplinary collaboration is essential (36, 37).

Like other chronic conditions, it is worth considering therapies with more than one mechanism or target. Alongside lifestyle-based interventions like diet and exercise, inadequate response to one treatment class must prompt combination therapy targeting complementary mechanisms (38). A recent study showed that liraglutide enhanced weight loss in sub-optimal MBS responders (39). Similarly, incretin and glucagon agonism (triple agonists like retatrutide), and amylin-incretin combined agonists are under trial (40). More such trials would help improve therapeutic choices and efficacy.

From a public health perspective, addressing the epidemic of obesity requires deliberate strengthening of preventive medicine as a discipline (1). The drastic increases in global food availability have outpaced human adaptation, yet not everyone in an abundant environment develops obesity; in developing countries, obesity is more prevalent in higher socio-economic classes, whereas the opposite is true in developed countries. Preventive medicine must have greater emphasis in medical education and training. Addressing clinical, behavioral, and social determinants early would help resolve metabolic disruptions before they become rooted into memory.

### Limitations

Our model has some inherent limitations that warrant acknowledgement. It is conceptual rather than empirical. Translating the model into practice will require empirical studies to test whether interventions at different hierarchical levels produce the expected durability of effect. Complementing models such as the satiety cascade, this framework offers a focused lens on hunger as a deviation from satiety, and on the temporal properties of metabolic signals. These distinctions define its scope; its purpose is to present a scaffold that highlights current gaps in understanding and organize potential therapeutic targets, rather than to offer a comprehensive explanatory system.

## Conclusion

In summary, obesity represents a complex interplay of genetic, developmental, and environmental factors that extend beyond simple weight gain. Our four-organ model of hunger and molecular hierarchy framework highlights how intracellular and extracellular molecules of different sizes act as scripts, playwrights, directors and actors within organ systems governing energy balance. Current incretin-based therapies provide remarkable but temporary relief, emphasizing the need for deeper exploration into the roots of metabolic programming and memory. Future research must aim toward disease modification. Clinical care must focus on better utilization of available interventions, develop preventive and personalized therapeutic goals, and integrate psycho-social determinants. Despite limitations, using these perspectives to understand obesity beyond reductionist fallbacks would allow the management of obesity at its roots, with strategies that are preventive, durable and accessible.

## Data Availability

The original contributions presented in the study are included in the article/supplementary material. Further inquiries can be directed to the corresponding author.
